# Biosurfactant production from marine hydrocarbon-degrading consortia and pure bacterial strains using crude oil as carbon source

**DOI:** 10.3389/fmicb.2015.00274

**Published:** 2015-04-07

**Authors:** Eleftheria Antoniou, Stilianos Fodelianakis, Emmanouela Korkakaki, Nicolas Kalogerakis

**Affiliations:** Biochemical Engineering and Environmental Biotechnology Laboratory, School of Environmental Engineering, Technical University of CreteChania, Greece

**Keywords:** biosurfactant, marine bacteria, *Alcanivorax*, rhamnolipid, sophorolipid, crude oil, bioaugmentation, *Paracoccus marcusii*

## Abstract

Biosurfactants (BSs) are “green” amphiphilic molecules produced by microorganisms during biodegradation, increasing the bioavailability of organic pollutants. In this work, the BS production yield of marine hydrocarbon degraders isolated from Elefsina bay in Eastern Mediterranean Sea has been investigated. The drop collapse test was used as a preliminary screening test to confirm BS producing strains or mixed consortia. The community structure of the best consortia based on the drop collapse test was determined by 16S-rDNA pyrotag screening. Subsequently, the effect of incubation time, temperature, substrate and supplementation with inorganic nutrients, on BS production, was examined. Two types of BS – lipid mixtures were extracted from the culture broth; the low molecular weight BS Rhamnolipids and Sophorolipids. Crude extracts were purified by silica gel column chromatography and then identified by thin layer chromatography and Fourier transform infrared spectroscopy. Results indicate that BS production yield remains constant and low while it is independent of the total culture biomass, carbon source, and temperature. A constant BS concentration in a culture broth with continuous degradation of crude oil (CO) implies that the BS producing microbes generate no more than the required amount of BSs that enables biodegradation of the CO. Isolated pure strains were found to have higher specific production yields than the complex microbial marine community-consortia. The heavy oil fraction of CO has emerged as a promising substrate for BS production (by marine BS producers) with fewer impurities in the final product. Furthermore, a particular strain isolated from sediments, *Paracoccus marcusii,* may be an optimal choice for bioremediation purposes as its biomass remains trapped in the hydrocarbon phase, not suffering from potential dilution effects by sea currents.

## Introduction

Chronic release of oil in the sea from numerous natural and anthropogenic sources poses a continuous-serious threat for the environment (Nikolopoulou and Kalogerakis, 2010). The majority of petroleum hydrocarbon input comes from natural seeps, while spillage from vessels or operational discharges have nowadays decreased significantly and, e.g., in North America only 1% of the oil discharges is related to the extraction of the oil. Approximately, 1.3 million tones of petroleum enters the marine environment each year (National Research Council (NRC) of the National Academies - Committee on Oil in the Sea, 2003; Diez et al., 2007; Ventikos and Sotiropoulos, 2014), while in the Gulf of Mexico alone after the Deep Horizon incident > 600,000 tones were released into the sea (International Tanker Owners Pollution Federation [ITOPF], 2013). Acute accidents such as the Deep Horizon result not only in increased public concern but also in mass mortality of marine and coastal life. Fortunately they are rare.

Oil pollution cleanup in marine environments with the use of biological means-bioremediation (Nikolopoulou and Kalogerakis, 2008; Nikolopoulou et al., 2013a,b), has emerged as a very promising ‘green’ alternative technology following first response actions (skimmers, boomers, fire, dispersion with chemical surfactants). Crude oil (CO) is biodegradable. Hydrocarbon-degrading bacterial consortia exist in nature and thrive in oil-polluted sites, while using petroleum hydrocarbons as source of carbon and energy for growth (Hassanshahian et al., 2012; McGenity et al., 2012; Thomas et al., 2014). The way hydrocarbon-degrading bacterial consortia and pure strains engineer their way into the oil spill for biodegradation is very complex and still under investigation. Bacterial cells produce a mixture of biosurfactant (BS) lipids with the help of which oil is dispersed into very fine droplets and thus the bioavailability of CO is increased.

Biosurfactants are surface-active compounds produced by microorganisms. They display a variety of surface activities (surface tension decrease from 72 to 30 mN/m Helvaci et al., 2004) that increase the bioavailability of organic pollutants, including CO components, and thus enhance biodegradation (Nguyen et al., 2008; Rahman and Gakpe, 2008; Whang et al., 2008; Banat et al., 2010, 2014; Nguyen and Sabatini, 2011; Randhawa and Rahman, 2014). BSs belong to a structurally diverse group of amphiphilic biomolecules with both hydrophilic and hydrophobic moieties. They generally are grouped either as low or high molecular weight BSs, the former consisting of glycolipids and lipopeptides and the latter of high molecular weight polymeric BS. Due to their biodegradability and low toxicity they are very promising for use in remediation technologies as an alternative to the synthetic surfactants (Nguyen et al., 2008). Microbial BSs can replace the currently used chemical surfactants that are more toxic in many applications, like combating oil spills, bioremediation enhancement, micro-extraction of PAHs, pharmaceutical products, and detergent industry (Nguyen et al., 2008; Banat et al., 2010; Nguyen and Sabatini, 2011). There is a need for ecologically friendly and biodegradable surfactants (ionic or non-ionic) for reliable environmental cleanup. Commercially viable BSs have to be economically competitive therefore the development of good microbial BS producing cultures is required (Banat et al., 2000, 2010, 2014; Nguyen et al., 2008; Rahman and Gakpe, 2008; Whang et al., 2008; Nguyen and Sabatini, 2011; Randhawa and Rahman, 2014). Nowadays BSs still have not been employed extensively in industry because of the high production cost.

Biosurfactant production challenges and solutions for increasing the production yield are very well presented by Banat et al. (2014). Problems that limit BS industrial production include the required renewable substrate media quantities, slow growth rate of organisms on the substrate, low yield and final product purification from substrate impurities. Although cost effective BS production is still a goal to be attained, other important issues currently under investigation include the development-isolation of BS producing microorganisms (consortia or strains), the fine-tuning of their production ability by changing their incubation conditions (temperature, time, nutrients) and/or substrate type toward achieving a high yield and the production of lipid mixtures with an attractive/desired structure.

The primary objective of this work was to investigate the BS production efficiency and quality of isolated consortia and pure strains (that have hydrocarbon-degrading capabilities) isolated from the sediment and water column of a hydrocarbon-contaminated marine area (Elefsina bay, Attica, Greece) with CO as sole carbon source. The fact that marine hydrocarbon degraders are often BS producers as well impelled us to investigate the BS production efficiency of specific marine hydrocarbon-degraders. The sampling and isolation of hydrocarbon degraders from Elefsina bay was part of the FP7 project ULIXES. In particular, the production of two BS types, rhamnolipids (RLs) and sophorolipids (SLs) by isolated consortia was investigated regarding the effect of incubation time, temperature, addition of nutrients N (as KNO_3_) and P (as KH_2_PO_4_), and finally the carbon source. Therefore, isolation, screening, detection and characterization techniques were used in order to evaluate/confirm the BS chemical composition. In addition, promising pure strains were also tested for their BS production ability. The effect of substrate on the RL yield of the best BS producing strain was investigated. In an attempt to explain the BS production yield, we try to answer the following questions: how the RL production yield by marine microbes compares to the critical micelle concentration (CMC)? How this relates to the oil degradation? What is the role - spatial distribution of BS in the process (emulsion, cell hydrophobicity increase)?

## Materials and Methods

### Sampling Locations

**FIGURE 1:**
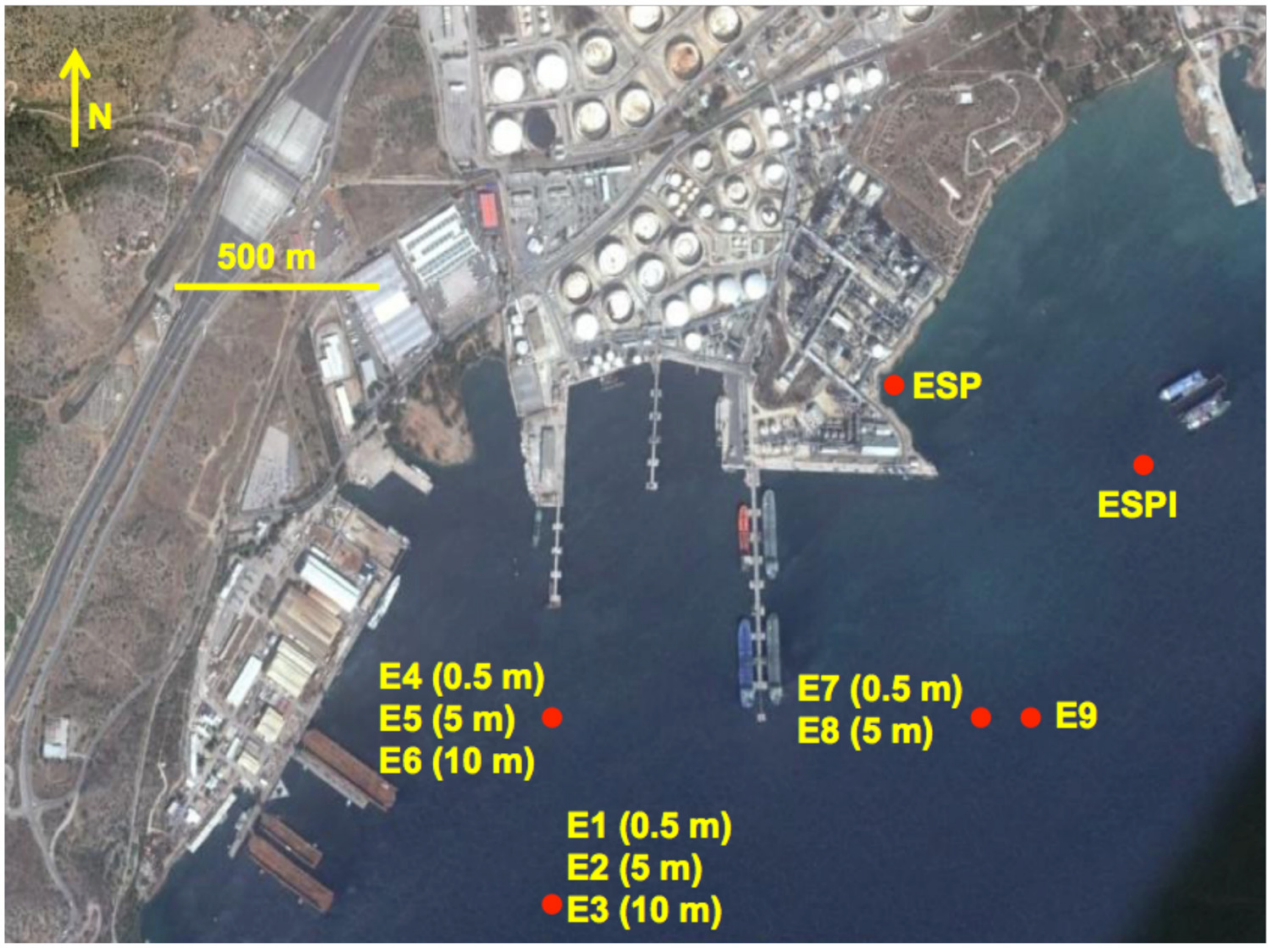
**The sampling campaign map. The locations of all the sampling sites are represented by red dots**. The code(s) of the consortia isolated from each sampling site is given next to each dot. For consortia isolated from the water column, i.e., consortia E1–E8, the depth of the water sampled for initial inoculation is given in parenthesis next to the consortium code. The image was captured and modified using Google Earth.

Seawater and sediment samples were collected from six locations in Elefsina bay, Attica, Aegean Sea as shown in **Figure 1**. Elefsina bay is a major industrial area, where among other industrial complexes, there are two large petroleum refineries. Due to several accidents in the past and the slow seepage of CO from old storage tanks, there is sufficient evidence of low chronic pollution in the area. The sampling campaign aimed to isolate consortia and strains from both the water column and the sediment, enhancing the probability to isolate different strains of interest. The samples were collected downstream of the local current direction (West-to-East). An additional sediment sample (ESP) was collected at the area where a small stream joins the bay.

### Preparation of Enrichment Cultures

Enrichment cultures were prepared by adding 10 ml of seawater or 10 g of sediment (for sediment samples) in 90 ml ONR7 (Yakimov et al., 1998), with the addition of 0.5% w/v filter-sterilized CO in 250 ml Erlenmeyer flasks. The cultures were incubated at 20°C, in an orbital incubator, agitated at 150 rpm. At each re-inoculation, 1 ml of culture from the early exponential phase was transferred to 99 ml of ONR7 medium. Plating count on marine agar for marine heterotrophs and OD measurements was carried out to establish reliable growth curves.

### Screening of Marine Consortia by the Drop Collapse Test for the Isolation of Pure Biosurfactant Producing Strains

The drop collapse test was performed according to (Youssef et al., 2004). Scoring was performed by setting sterile deionised water as a negative control and a 10^-4^ dilution of “S-200 oil-gone” commercial BS solution (IEP Europe S.L., Madrid) as a positive (+++) control and comparing the diameter of droplets from the examined cultures. Scoring, of “-” to “+++” was performed by comparing the diameter of the droplet (X) to that of the water droplet (Y) and the positive control (Z). A “-” score was given if X ≤ Y whereas a “+++” score was given if X ≥ Z. Finally, a “+” score was given if Y < X ≤ (Z-Y)/2 and a “++” if (Z-Y)/2 < X < Z.

For the drop collapse test, consortia were incubated at 14°C (in an effort to mimic the original aquatic habitat temperature) for 6 weeks in ONR7/CO 0.5% w/v as a sole carbon source. Re-inoculations were performed weekly. The drop collapse test was performed once every week, just before the re-inoculation.

### Initial Community Screening of Biosurfactant Producing Consortia by Pyrotag Sequencing

Total genomic DNA was extracted according to (Moore et al., 2004). DNA yield and quality was determined by agarose gel electrophoresis of 5 μl of DNA extract. DNA extracts were stored at 4°C until use.

PCR and pyrosequencing were performed in Research and Testing Laboratory (Lubbock, TX, USA) on an FLX Titanium platform, for the V4 hypervariable region of the 16S rRNA gene using primers 515F (5′-GTGCCAGCMGCCGCGGTAA-3′) and 806R (5′-GGACTACHVGGGTWTCTAAT-3′) which are known to have reduced bias and cover a wide range of bacterial and archaeal phyla (Kuczynski et al., 2012). Noise filtering and chimera removal (using the AmpliconNoise package Quince et al., 2011), operational taxonomic unit (OTU) clustering (at 97% similarity, using uclust Edgar, 2010), OTU table construction, Good’s coverage index estimation (Good, 1953) and phylogenetic assignments (comparing against the latest Greengenes database release McDonald et al., 2012 with uclust) were performed in QIIME v1.8 (Caporaso et al., 2010). The samples for the whole project have been deposited in the NCBI short read archive (SRA) database under the BioProject accession number PRJNA190077.

### Isolation and Characterization of Pure Biosurfactant Producing Strains

Hundred μl of each mixed culture taken at the early stationary phase were initially spread on Zobell marine agar or ONR7 agar/CO 0.5% w/v in triplicates, at a dilution of 10^-4^ and 10^-6^. Colonies of distinct morphology were carefully picked and reinoculated on the same medium. Single colonies were then picked and the growth of each isolated strain was tested in both ONR7/CO 0.5% w/v and marine broth at 14°C.

For the characterization of pure isolates, 200 μl of each culture was centrifuged for 5 min at 10000 g and after aspiration of the supernatant, the resulting pellet was incubated for 15 min at 95°C with 50 μl STE buffer (100 mM NaCl, 10 mM Tris-HCl pH 8, 1 mM EDTA pH 8) and 1 μl of the resulting solution was used as a template for PCR. PCR was prepared in a laminar flow chamber under aseptic conditions and performed in an Eppendorf Mastercycler gradient. Negative controls (autoclaved ultra-pure water) were used in every reaction. A ∼1500 bp fragment of the bacterial 16S rRNA locus was amplified using the universal bacterial primers 27F (5′-AGAGTTTGATC(AC)TGGCTCAG-3′) and 1492R (5′-ACGG(CT)TACCTTGTTA CGACTT-3′; Weisburg et al., 1991), as described in Fodelianakis et al. (2014). Quantity and quality of the PCR products were evaluated by agarose (1.2%) gel electrophoresis. PCR products were then purified using the Nucleospin Gel and PCR cleanup (Machery-Nagel) commercial kit. Purified PCR products were sequenced in StarSEQ GmbH, Mainz, Germany from both the forward and reverse primers. The overlapping sequence (∼890 bp) was compared against the NCBI nr and 16S database using the BLAST algorithm in order to find the closest relative and the closest described species respectively.

### Biosurfactant Production

#### Cultivation

The isolated BS producing consortiums or pure strains were inoculated in 200 ml of ONR7 medium supplemented with 0.5%w/v carbon source and incubated on an orbital shaker at 150 rpm for 5–6 and 10–12 days at different temperatures. The nutrients added were KNO_3_ and KH_2_PO_4_ until the final ratio of C:N:P was equal to 100:10:1. The origin of the CO used as the carbon source for the experiments was Kazakhstan. The isolated single strains were cultivated at 14°C (aquatic habitat temperature) for 6 and 12 days, in 200 ml ONR7 medium with 0.5% w/v CO as the carbon source. To test RL production of strain E8Y (see Results) under different carbon sources, the following media were used: (i) Glucose 0.5% wt, (ii) glucose 0.25% wt and CO 0.25% wt, (iii) glucose 0.25% wt and heavy CO fraction (asphaltene-aromatics fraction with less than 7% saturates) 0.25% wt and (iv) 0.5% wt CO.

#### Liquid-Liquid Extraction

The extraction of crude BS extract, free from the aqueous culture medium, was performed by liquid–liquid extraction. RLs were extracted with equal volume of ethyl-acetate, after centrifugation at 13,000 ×*g* for 15 min at 4°C to remove the bacterial cells and acidification of the culture medium at PH = 3 with 6 N HCl (Smyth et al., 2010), while SL extract was obtained from the whole culture using three times equal volume of ethyl-acetate (Nuñez et al., 2001). Anhydrous sodium sulfate was added to the ethyl acetate layer to remove residual water, filtered, was collected in a round-bottom flask and connected to a rotary evaporator to remove the solvent. The process yielded a viscous honey-colored BS.

#### Biosurfactant Purification

Purification of the produced BS crude extract was conducted using Silica Gel Column Chromatography. Silica gel 60 (240–425 mesh) was the stationary phase in all cases. Neutral lipids, Rha-C_10_-C_10_ and Rha-Rha-C_10_-C_10_ purified fractions were obtained using CHCl_3_:CH_3_OH (50:3 v/v), CHCl_3_:CH_3_OH (50:5 v/v) and CHCl_3_:CH_3_OH (50:50 v/v) as the mobile phase, respectively. Acidic and lactonic types of SLs were obtained using CHCl_3_:CH_3_OH (98:2 v/v), CHCl_3_:CH_3_OH (83:17 v/v), CHCl_3_:CH_3_OH (71:19 v/v) and CHCl_3_:CH_3_OH (60:40 v/v) as the mobile phase (Smyth et al., 2010; third and fourth fraction carried the different types).

### Biosurfactant Detection-Characterization

A RL mixture of Rha-C_10_-C_10_ and Rha-Rha-C_10_-C_10_ (Aldrich Chemistry, R-95 RL 95%) was used as standard to compare the RLs produced.

#### Thin Layer Chromatography (TLC)

Biosurfactant detection was performed on the crude extract by TLC on pre-coated silica gel of standard 20 × 20 Kiesel-gel 60 F254 Merck plates using the appropriate solvent system and visualization agent for each BS. In the case of RLs, the solvent system used was chloroform : methanol : acetic acid (65:15:2, v/v/v), the spray reagent was antrone (Smyth et al., 2010), while SLs were detected via chloroform : methanol : water (65:15:2, v/v/v) and the development agent was *p*-Anisaldehyde (100°C for 5 min; Asmer et al., 1988).

#### Fourier Transform IR Spectroscopy (FT-IR)

Infrared spectroscopy is a simple method for structure analysis. Samples were liophilized and milled with KBr to form a uniform capsule and were characterized via FT-IR spectroscopy on a Perkin Elmer 2000 FTIR spectrometer operated in the absorbance mode at a resolution of 4 cm^-1^.

#### Mass Spectrometry (LC-MS)

Rhamnolipid mixtures were separated and identified by liquid chromatography coupled to mass spectroscopy using an Agilent Technologies 6110 quadrupole LC–MS (Smyth et al., 2010). Samples were prepared with ACN:H_2_O (80:20 v/v; LC grade) with a concentration of 10 mg/l and 100 μl of the same was injected into a C_18_ (150mm × 2.1mm × 5 μm) column. The LC flow rate was 0.25 ml/min. For mobile phase, an acetonitrile–water gradient was used starting with 40% of acetonitrile for 4 min, followed by 40–90% acetonitrile in 20 min then return to the initial condition in 6 min. Ammonium acetate buffer was added.

## Results

### Mixed Culture Screening and Pure Strain Isolation

Eleven enrichment cultures, namely E1-E9, ESP and ESPI were obtained from the respective seawater and sediment samples. Those cultures were then screened by the drop collapse test for BS production. As presented in **Table 1**, consortium E8 achieved the highest overall scores within the given time period, followed by consortium E4. An additional consortium, namely EB8, was subsequently created from the E8 consortium by re-inoculating material only from the oil-culture interface, to further test if the BS production could be enhanced further by these isolates (adaptation of the MATH test isolation method).

**Table 1:** Drop collapse test results for each consortium.

Sample	Origin	W1	W2	W3	W4	W5	W6
E1	Water	-	-	-	-	+++	-
E2	Water	+	+	+	+	+	++
E3	Water	-	+	-	-	+	-
E4	Water	++	++	+	++	++	++
E5	Water	-	-	-	++	+++	-
E6	Water	-	-	+	-	+	-
E7	Water	-	-	-	-	-	-
E8	Water	++	++	++	+++	++	++
E9	Sediment	+	-	+	++	-	++
ESP	Sediment	-	+	+	-	+	+
ESPI	Sediment	-	-	+	+	+	+

*W1, W2, W3, W4, W5, and W6 stand for scores on weeks 1, 2, 3, 4, 5, and 6 respectively*.

Subsequently, the community structure of consortia E8, EB8 and E4 was examined with 16S rRNA gene pyrotag sequencing. Consortium E9 was also included in this analysis due to its different origin (sediment) and therefore its potential to contain different BS producing strains. Good’s coverage estimates ranged between 0.97 and 0.99, indicating that the sampling depth was enough for an adequate description of the bacterial diversity of each of the examined consortia.

**FIGURE 2:**
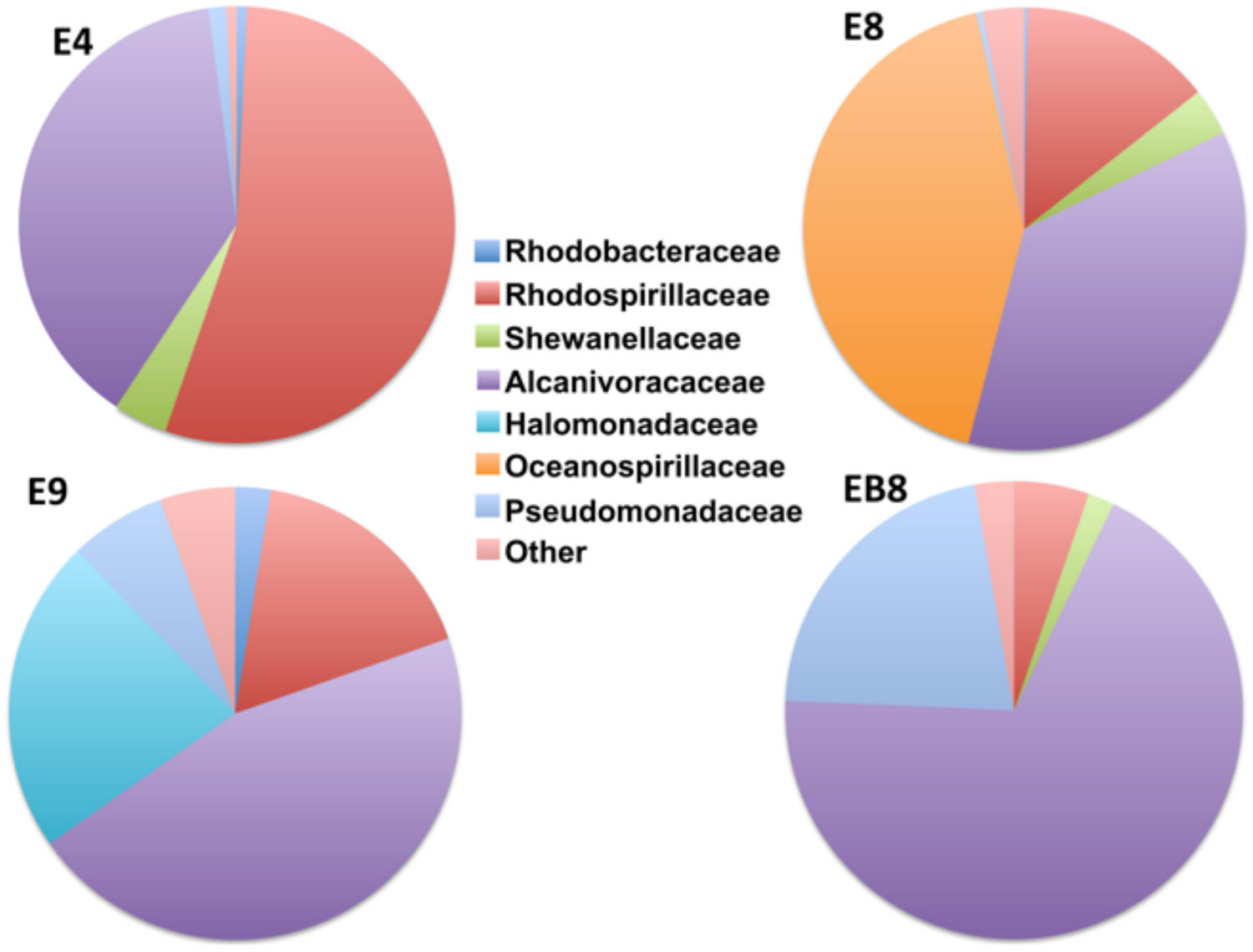
**The distribution of the bacterial Families among the screened consortia**. The “Other” category stands for reads for which assignment down to family level was not possible.

Phylogenetic analysis of the pyrotag reads revealed the presence of seven families among the examined consortia; Rhodobacteraceae (0.07–2.5% per sample reads), Rhodospirillaceae (5.3–54.5% per sample reads), Shewanellaceae (0–4% per sample reads), Alcanivoracaceae (36.2–68.5% per sample reads), Halomonadaceae (0–22.4% per sample reads), Oceanospirillaceae (0–42.4% per sample reads) and Pseudomonadaceae (0.4–21.6% per sample reads; **Figure 2**). For some reads (0.8–5.4% per sample) phylogenetic assignment down to the family level was not possible (**Figure 2**).

Upon further examination we found that within-family evenness was very low; the majority of reads within each family could be assigned to a single OTU. These OTUs were the most probable to be isolated and tested for their ability to produce BSs. The representative sequences of the most abundant OTUs within each family were then compared against the NCBI 16S database using BLAST in order to find the closest cultivated representative to each abundant OTU. **Table 2** summarizes the distribution of reads within each of the seven families and the BLAST results for the most abundant OTUs within each family.

**Table 2:** Within-family read distribution and closest relative of the most abundant OTUs.

Family	# of within-family OTUs	% of the most abundant OTU	Closest Relative of the most abundant OTU (% of similarity)
Rhodobacteraceae	3	91	*Roseovarius crassostreae* (99)
Rhodospirillaceae	18	96	*Thalassospira lucentensis* strain QMT2 (98)
Shewanellaceae	3	98	*Shewanella frigidimarina* strain NCIMB 400 (99)
Alcanivoracaceae	29	95	*Alcanivorax borkumensis* SK2 (99)
Halomonadaceae	3	93	*Halomonas marina* (99)
Oceanospirillaceae	11	97	*Marinomonas vaga* strain 40 (99)
Pseudomonadaceae	3	96	*Pseudomonas pachastrellae* strain KMM 330 (99)

### Pure Strain Isolation and Biosurfactant Production Screening

As shown in **Table 2**, the number of possible isolates obtained from consortia E4, E8 and E9 was limited, as these were dominated by a handful of dominant strains. Thus, the chance of isolating strains other than those was statistically disfavoured. In order to increase the total number of possible BS producers, two more sediment samples from Elefsina, namely ESP and ESPI, were included. Isolation and purification of single strains was subsequently performed as described in Experimental Procedures.

Fifty pure cultures were obtained in total and their taxonomy was identified (see Experimental Procedures). Twelve of these strains were actually different. The ability of each different strain to grow in rich (marine broth) and minimal (ONR7a) medium with CO 0.5% w/v as a sole carbon source was then examined. All taxonomically different isolates were consequently screened for BS production by the drop collapse test in the medium(s) were growth was possible. The isolated strains’ phylogenetic identity, growth ability and drop collapse test scores are shown in **Table 3**. Strains E8Y, E4D, E4F (*Alcanivorax borkumensis* SK2) in ONR7a/CO 0.5% w/v and ESP-A (*Paracoccus marcusii*) in ONR7a/CO 0.5% w/v achieved the highest scores. BS production of these strains was then quantified.

**Table 3:** Code, phylogenetic identity, growth ability and drop collapse test results of the isolated pure strains.

Strain code	Closest Relative	Growth in marine broth	Growth in ONR7a/ crude oil 0.5% w/v	Drop collapse test in marine broth^1^	Drop collapse test in ONR7a/ crude oil 0.5% w/v^1^
XP2	*Pseudomonas pachastrellae* strain KMM 330	Yes	No	+	n/a
XP3	*Marinomonas vaga* strain 40	Yes	No	-	n/a
XP4	*Thalassospira lucentensis* strain QMT2	Yes	No	-	n/a
XP5	*Thalassospira lucentensis* strain QMT2	Yes	No	-	n/a
XP6	*Roseovarius crassostreae*	Yes	No	+	n/a
E8Y	*Alcanivorax borkumensis* SK2	No	Yes	n/a	+++
E4D	*A. borkumensis* SK2	No	Yes	n/a	+++
E4F	*A. borkumensis* SK2	No	Yes	n/a	+++
ESP-A	*Paracoccus marcusii*	Yes	Yes	+	+++
ESP-C	*Sulfitobacter pontiacus* ChLG-10	Yes	No	+	n/a
ESPI-G	*Pseudoalteromonas agarivorans* strain KMM 255	Yes	No	+++	n/a
ESP-B	*Paracoccus carotinifaciens* strain E-396	Yes	Yes	-	-

*^1^The median score value of three biological replicates (separate cultures) is reported*.

### Biosurfactant Production by Mixed Bacterial Community – Effect of Time, Temperature, and Carbon Source

**FIGURE 3:**
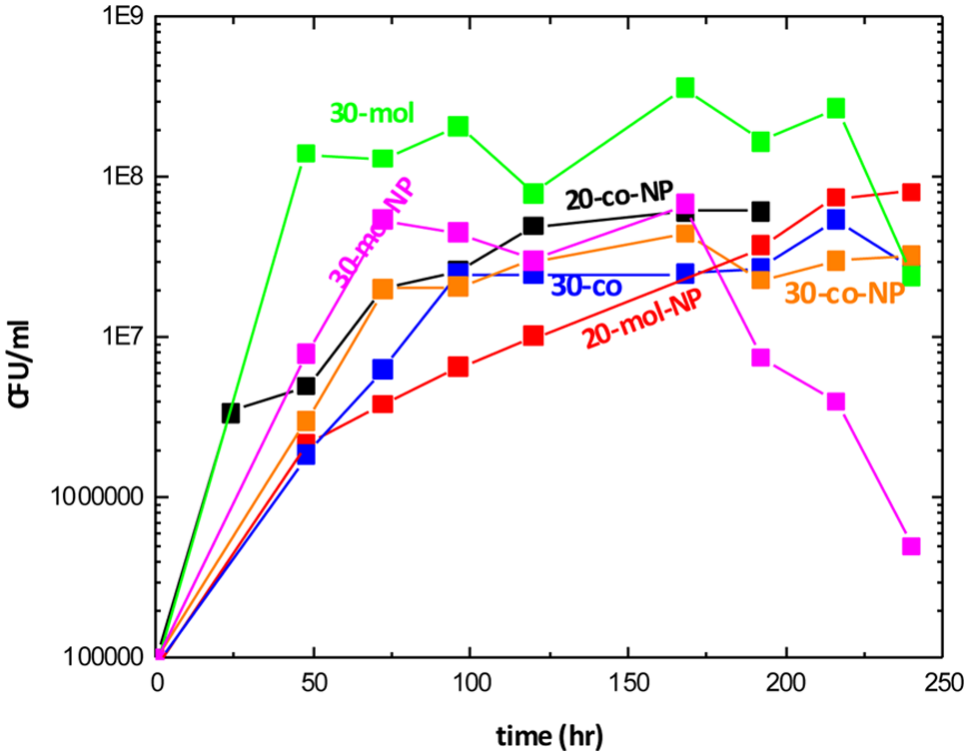
Growth curves, colony forming units vs. time, under different conditions. (where mol, molasses and crude oil; co, crude oil; NP, additional nutrients and the number in the legend represents temperature).

EB8 consortium was isolated as the most promising BS producing mixed culture based on the drop collapse test. The EB8 isolated community growth curves under the different incubation conditions are shown in **Figure 3**. All samples exhibited the same trend, reaching the stationary phase within 48 h. Bacterial growth reached the highest level when molasses and CO were used as a carbon source at 30°C, without addition of N and P sources.

**FIGURE 4:**
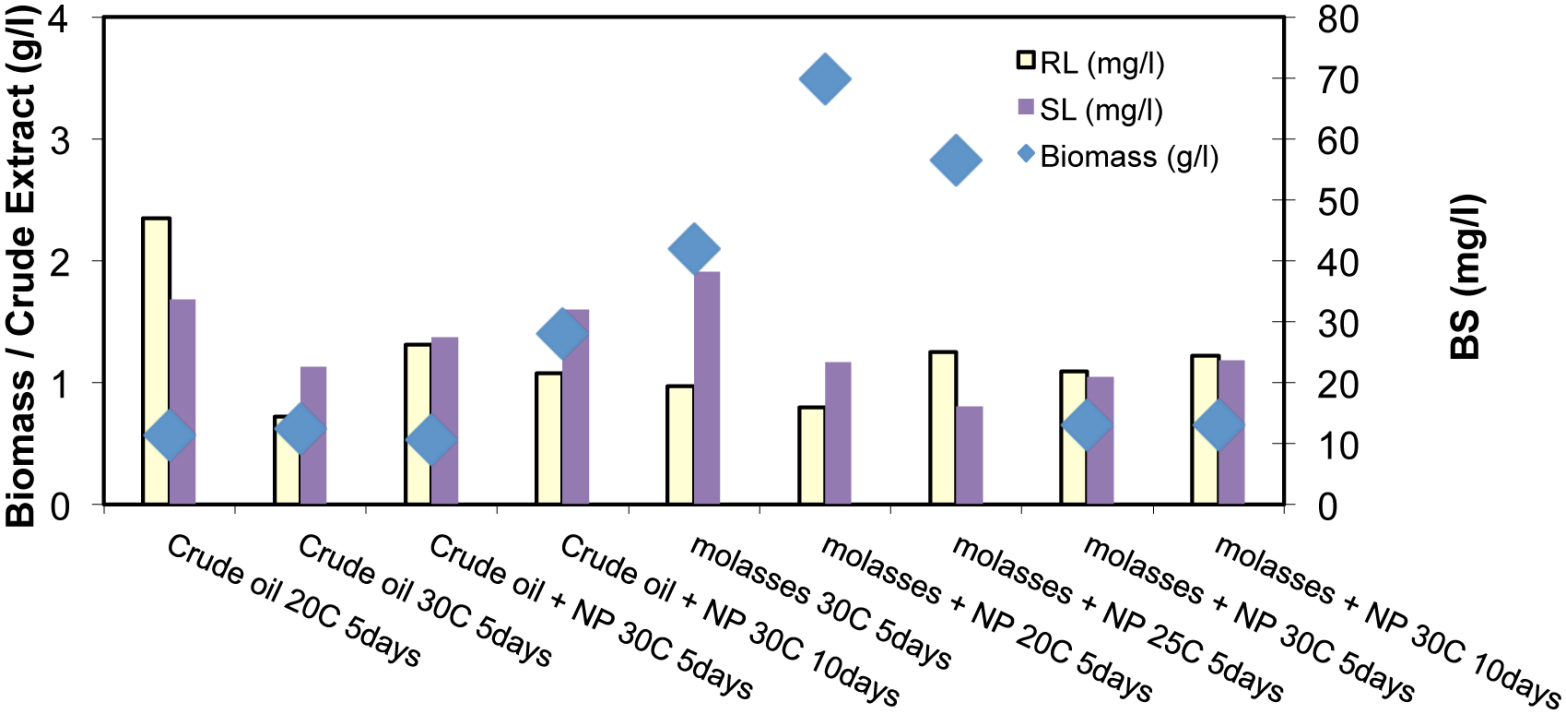
Biomass concentration (g/l), and purified Rhamnolipid (RL) and Sophorolipid (SL; mg/l) for the different inoculation conditions and substrates of BE8 consortium (where NP means additional nutrients; and the number in the legend represents temperature)

In **Figure 4** the BS production from BE8 isolated consortium is presented for different incubation conditions. We observe that RL concentration remained constant and low at ∼20 ± 10 mg/l in the culture broth, independent of incubation conditions. The BS production yield of BE8 was about double compared to the BS production yield of the E8 consortia (∼10 mg/l of purified RL), at 20°C with CO as carbon source. Addition of molasses led to increased biomass production of BE8 that decreased as temperature increased from 20 to 30°C but did not enhance BS production. In the presence of CO as carbon source, biomass concentration remained constant at all temperatures and approximately at 0.5 g/l. Nutrients addition (N and P) did not have a significant effect on biomass growth or BS concentration.

Sophorolipid production is shown to be in the same range as that of the RLs (20 ± 10 mg/l). Similar to RLs, the production of SLs remained constant over time and does not depend on the carbon source, temperature or biostimulation with N and P.

### Biosurfactant Production by Single Strains

**FIGURE 5:**
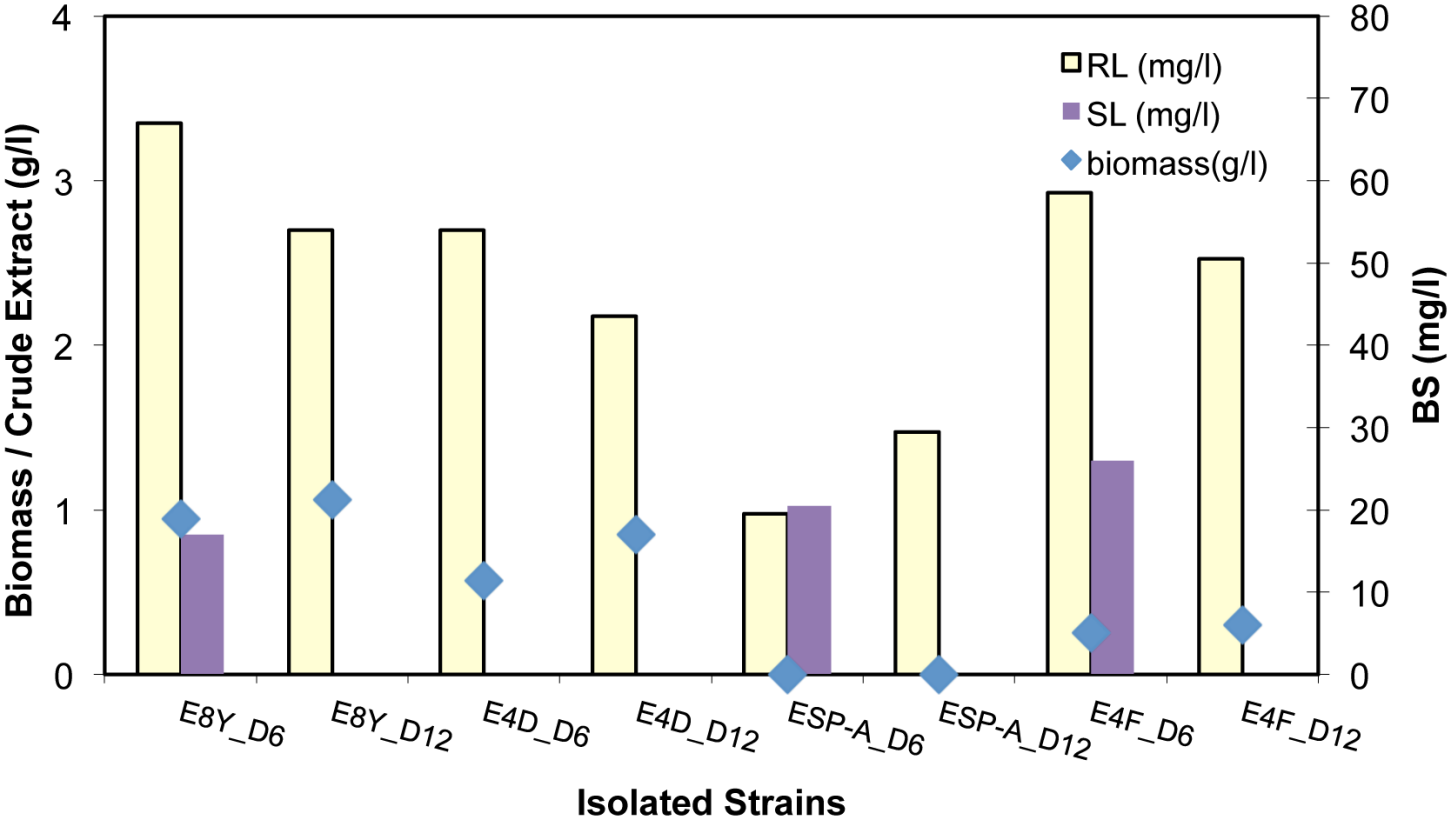
Biomass (g/l), purified RL and SL concentration (mg/l) from the cultivation of strain E8Y, E4D, ESP-A, E4F for 6 and 12 days

Both RL and SL BS production using the isolated strains E8Y, E4F, E4D, and ESP-A, were investigated, focusing now on the RLs production. The results are shown in **Figure 5**. Interestingly, we observed that biomass remained more or less constant from day 6 to day 12 while RL production yield was also in the same levels for the case of E8Y, E4D, and E4F strains, i.e., 50 ± 10 mg/l (more than 100% increase in the production of purified RL). Strain E4F exhibited the highest production yield of BS at the lowest biomass content. Strains ESP-A, E4F, and E8Y were further tested for SL production. A low production yield of 20 mg/l in all cases was observed.

The biomass of strain ESP-A remained within the CO phase and did not precipitate by centrifugation. The corresponding BS production was low compared to the rest of the other three isolated strains; however, this strain may have significant advantages for “sustainable” bioaugmentation in the open sea environment (no dilution by seawater currents).

It must be noted that the purified BS production yield from the crude BS extract was low of the order of 0.01–0.03 g BS/g crude extract, when CO was used as carbon source. This is a significant disadvantage of the use of CO as carbon source as it contaminates the crude extract with CO impurities, and a purification step with column chromatography becomes necessary.

### Rhamnolipid Production by Strain E8Y – Effect of Carbon Source

In order to understand the mechanism that drives BS producing strains toward BS production, and hence enable us to fine-tune the production yield, we investigated BS production using different carbon substrates as energy source.

**FIGURE 6:**
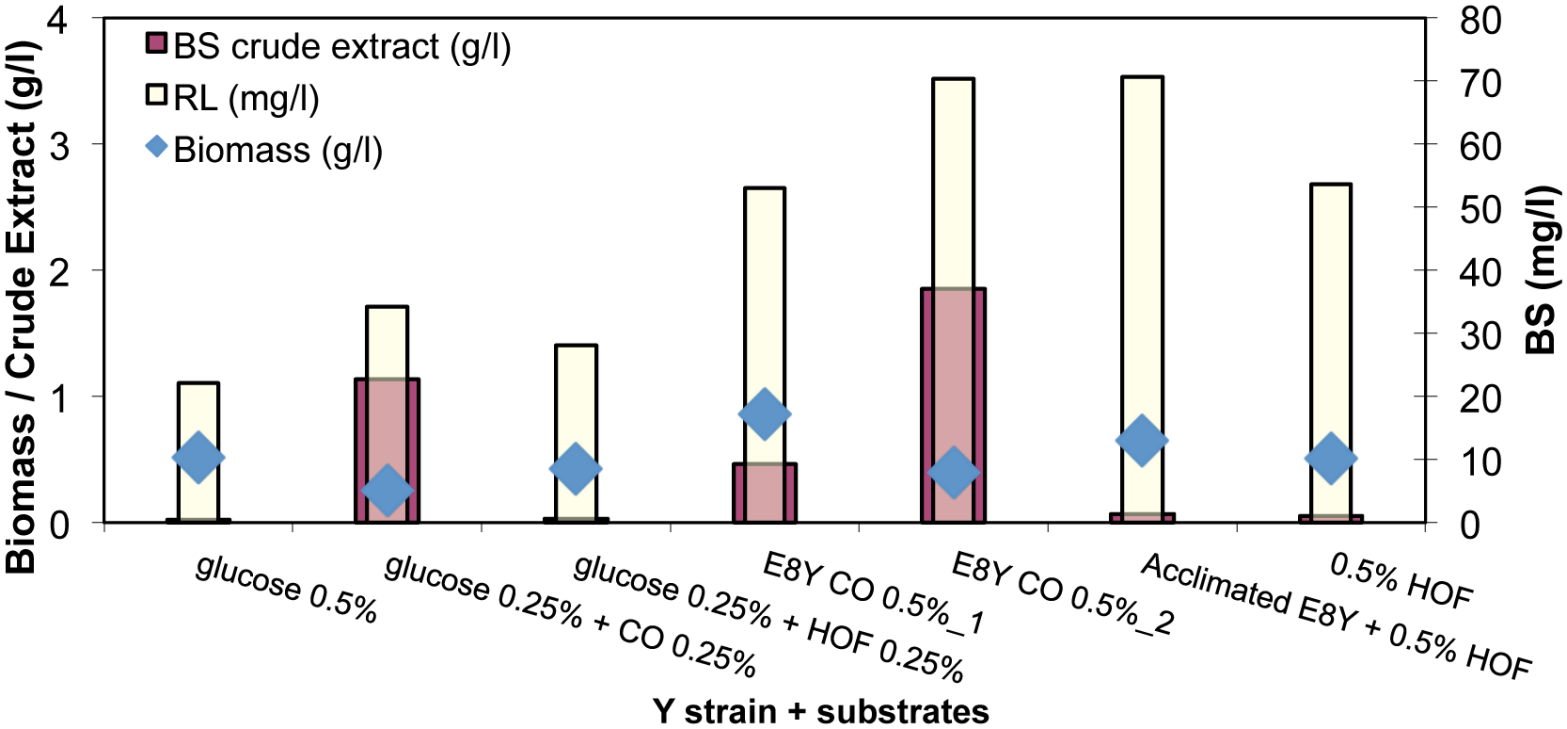
Biomass (g/l), BS crude extract (g/l), purified RL (mg/l) concentration produced by the cultivation of strain E8Y for 12 days with substrates pure or mixtures of glucose, crude oil (CO), and heavy oil fraction (HOF). In sample E8Y CO 0.5% wt_1 BS was extracted by the culture without the oil phase. In sample E8Y CO 0.5% wt_2 BS was extracted by the whole culture.

As shown in **Figure 6**, strain E8Y, a marine bacterial isolate, produced low concentrations of BS 50 ± 20 mg/l in the presence of the different carbon sources. In the absence of hydrocarbons, production of RLs decreased in half. Furthermore, the sample with CO as carbon source was made in duplicate and the BS concentration, extracted from the whole culture (0.5% wt CO-2), was compared to the one extracted by the culture without the oil phase (0.5% wt CO-1). A small difference (∼10 mg) is observed with the whole culture giving a better yield (∼70 mg purified BS/l). In addition, the culture with the heavy oil fraction (HOF) was made also in duplicate, one with E8Y strain (E8Y + 0.5% wt HOF) and a second one with acclimated to the HOF substrate E8Y strain (acclimated E8Y + 0.5% wt HOF). A better yield is observed when the acclimated strain was used (∼70 mg/l compared to 55 mg/l). Biomass remained low in all cases in the range 0.5 to 1 g/l.

When the heavy CO fraction was used, BS production was the highest (∼70 mg/l) and comparable to the BS concentration produced when CO was used as carbon source (50 ± 20 mg/l). This result is quite interesting because the HOF remains at the surface of the culture broth at all times and the extraction of the BS does not involve oil hydrocarbons as the substrate can be readily removed from the culture medium. The hydrocarbon-free culture medium is extracted without substrate impurities and the BS product is easily purified and produced.

### Characterization of the Purified Biosurfactants

All samples were characterized by the following techniques in order to confirm/identify the BS characteristics of the generated product.

#### Thin Layer Chromatography

**FIGURE 7:**
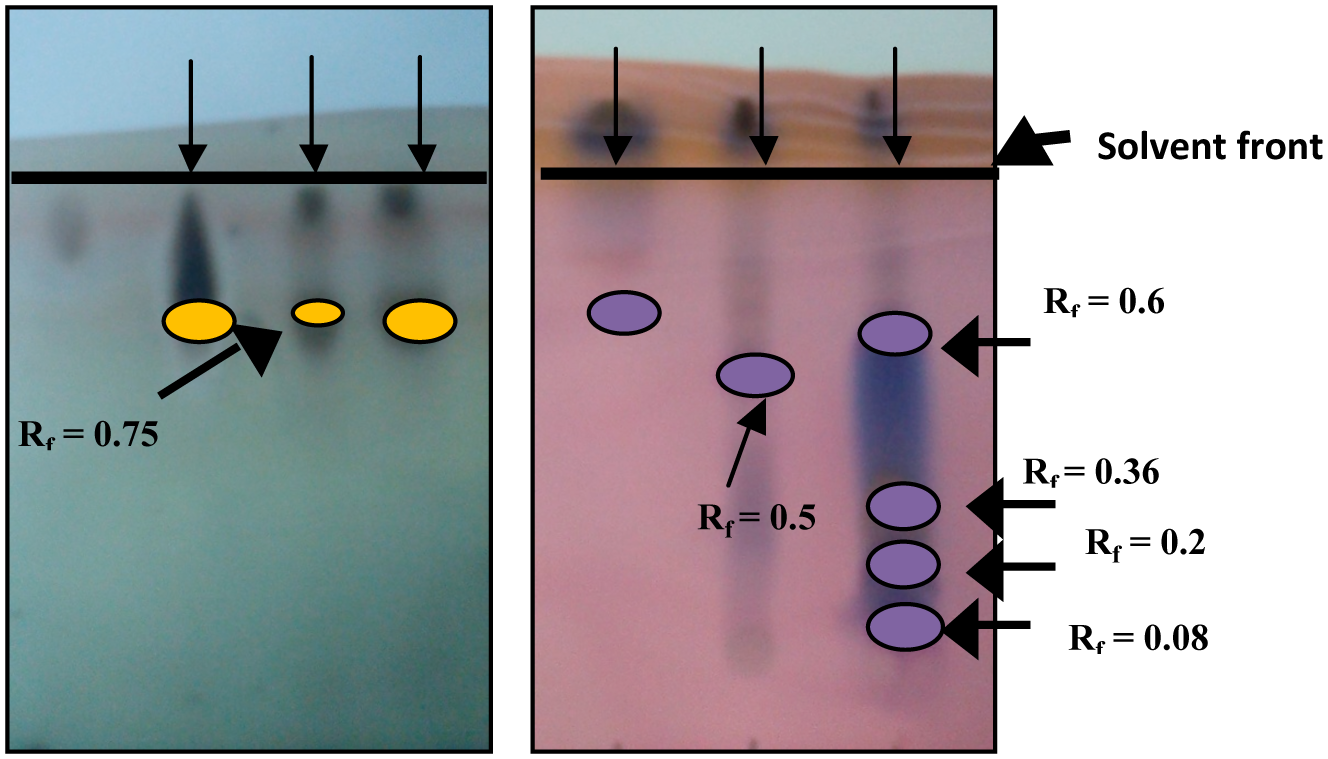
Rhamnolipids (left) and Sophorolipids (right) TLC detection

Thin layer chromatography results confirmed the presence of RLs and SLs in the crude extract and the purified BS products (**Figure 7**). Thin-layer chromatogram of isolated RL had an R_f_ value of 0.74 (band/solvent front ratio). SLs showed five prominent bands of R_f_ values of 0.08, 0.2, 0.36, 0.5, and 0.6 which compare with those published by Asmer et al. (1988).

#### FT-IR Measurements

**FIGURE 8:**
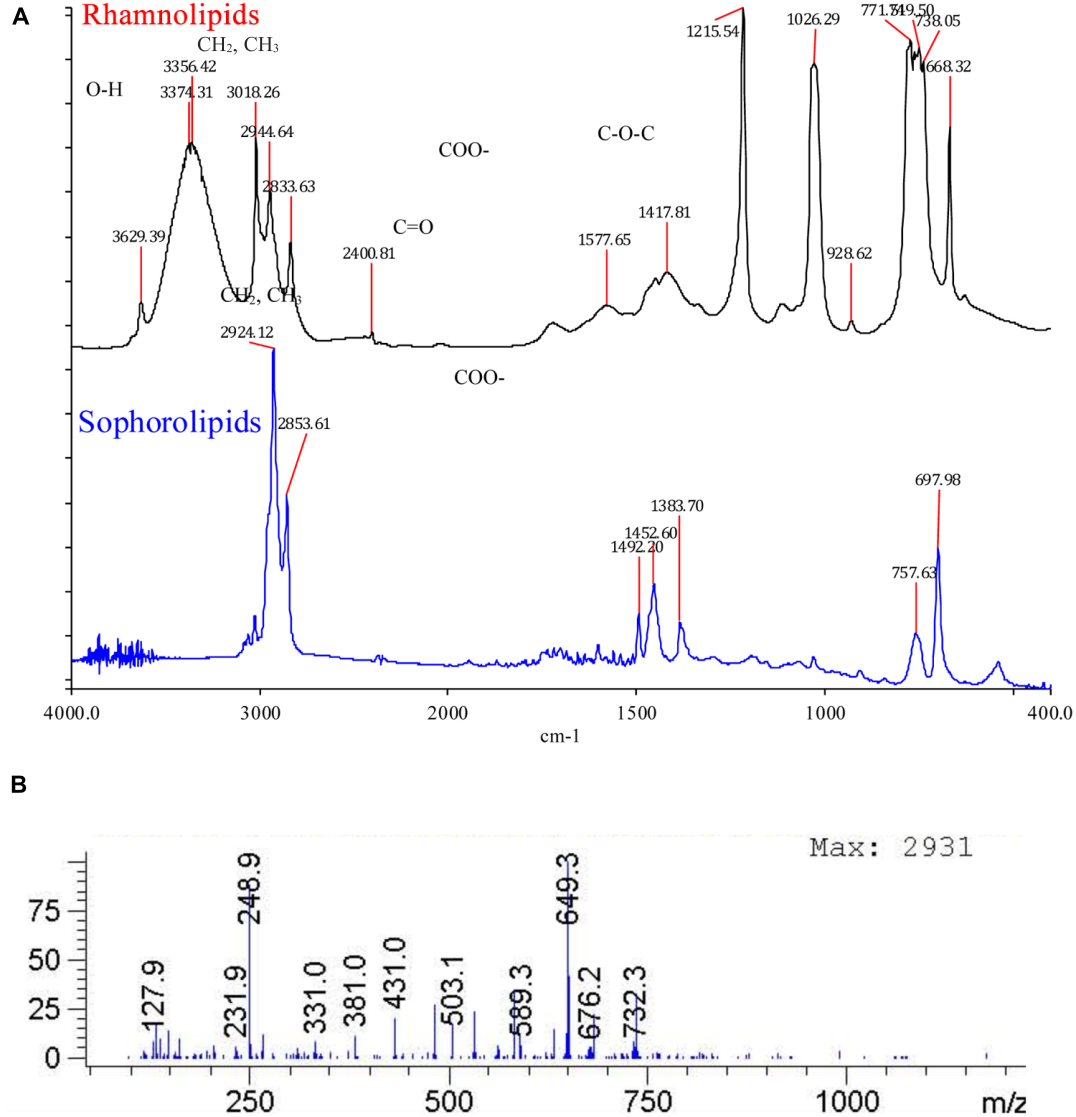
**(A)** Fourier transform infrared (FT-IR) spectrum of isolated rhamnolipids and sophorolipids. The characteristic peaks at 2930 (CH_2_, CH_3_), 2830–2850 (CH_2_, CH_3_), 1725 (C = O), 1450 (COO^-^), 1300-1100 confirm the presence of glycolipid type biosurfactant. **(B)** Rhamnolipids Rha-Rha-C10-C10 and Rha-C10-C10 detected with pseudomolecular ion being 649 and 503 respectively.

Fourier transform infrared characteristic peaks at 3350, 2930, 2860, 1400, 1638, confirmed the presence of glycolipid type BSs (**Figure 8A**). FT-IR is a powerful tool to study the different forms of BSs. **Figure 8A** shows FT-IR spectrums of the purified RL and SL. In the entire spectrum, similar absorption arising from the O-H stretching vibrations occurs in the region of 3350 cm^-1^. The carbonyl functional group (C = O) had a peak in the region of 1744 cm^-1^. The asymmetrical stretching (VasCH2) and symmetrical stretching (VasCH2) of methylene occurs at 2926–2930 and 2850–2860 cm^-1^, respectively. The stretch of C-O band of C (-O)-O-C in acetyl esters appears at 1247 cm^-1^ (RL curve). The band at 1445 cm^-1^ that corresponds to the C-O-H in plane bending of carboxylic acid (–COOH; Silverstein and Webster, 1998).

#### Detection of Rhamnolipids with LC-MS

Rhamnolipids Rha-Rha-C_10_-C_10_ and Rha-C_10_-C_10_ were detected with pseudomolecular ion being 649 and 503 respectively (**Figure 8B**).

## Discussion

### The Biosurfactant Production Capacity of Marine Microbial Populations

The community structure of the four screened hydrocarbon degrading mixed consortia (E4, E8, BE8, E9) was rather simple. Overall, *Alcanivoracaceae* was the most abundant bacterial family with *A. borkumensis SK2* strain being the dominant strain. That specific strain is very well described in the literature (Yakimov et al., 1998; Golyshin et al., 2003; Schneiker et al., 2006) and it is one of the major players in hydrocarbon degradation in the water column; being commonly found in enrichment cultures and contaminated areas (Yakimov et al., 1998; Golyshin et al., 2003; Schneiker et al., 2006). Members of the *Rhodobacteraceae, Rhodospirillaceae, Halomonadaceae, Oceanospirillaceae, Pseudomonadaceae,* and *Shewanellaceae* families have also been reported to encompass oil-degraders and BS producers (Cui et al., 2008; Fredrickson et al., 2008; Mnif et al., 2009; Raaijmakers et al., 2010; Jiménez et al., 2011; Kostka et al., 2011; Ibacache-Quiroga et al., 2013). However, the single strains isolated from these families in this study did not show significant BS production (drop collapse test). This fact may indicate that these strains were acting mainly as hydrocarbon degraders and not BS producers in the mixed cultures. On the contrary, the three tested *A. borkumensis SK2* strains were found to produce BS, indicating that those were the main producers in the mixed cultures. These findings highlight the functional plasticity of microbial communities in the presence of hydrocarbon contaminants; hydrocarbon degradation is not a trait “bound” by taxonomy, as the cassettes of genes that encode for hydrocarbon degradation are typically found within mobile genetic elements and can be transmitted horizontally (Top and Springael, 2003). Thus, a microbial community can adapt and regulate its functions depending on the community composition and the presence of different substrates. Despite the above, there is a “core set” of bacteria (such as members of the *Alcanivorax*, *Marinobacter*, and *Cycloclasticus* genera) that are commonly isolated from marine hydrocarbon-degrading consortia as they are obligate oil-degraders (Yakimov et al., 2007).

The EB8 consortium that was created using material from the oil-water interface of E8 consortium, consisted mainly of *Alcanivoracaceae* and *Pseudomonadaceae* as opposed to the “mother” E8 consortium that also contained *Oceanospirillaceae* in a high proportion. That indicates that members of the latter family were mainly present in the planktonic form in the water phase of the culture. A mixture of mono-, di-rhamnolipid and SL glycolipids was produced by the EB8 consortium. BS concentration in all cases was low, in the range of 20 ± 10 mg/l (comparable to the concentration produced by the mixed consortia E8 at 20°C with CO as the carbon source) and remained constant over time (after the stationary phase is reached). A temperature increase from 20 to 30°C or addition of glucose (molasses) to the carbon source had no significant effect on the BS production even though biomass increased (from 0.5 to 3.5 g/l) in the presence of molasses and temperature increase. Biostimulation with N and P had no major effect on the biomass or the BS concentration. To the best of our knowledge a constant BS production by hydrocarbon degrading-BS producing single strain or consortia regardless of biomass and culture conditions has not been reported as of yet. Rahman et al. (2003) report BS production yield that is not related to the biomass concentration (high yield-low biomass or the opposite). In this work the BS production yield depends on the amount of the non-soluble substrate. In the case of marine *Bacillus* sp. the BS product to biomass ratio varies with substrate variation (Mukherjee et al., 2008). This was also observed in this work.

There are only few reports available about production of BSs by bacterial consortiums isolated from hydrocarbon contaminated soil or marine water column (Rahman et al., 2003; Darvishi et al., 2011; Trejo-Castillo et al., 2014) and even fewer that at the same time use CO as the carbon source. In particular, Rahman et al. (2003) and García-Rivero et al. (2007) used crude and diesel oil as sole carbon source respectively. Furthermore, the consortium they used is a mixed culture comprising from the best single strains that had a specific characteristic, e.g., good BS production or hydrocarbon degradation as opposed to ours that has been subcultured directly from environmental samples. Darvishi et al. (2011) and Rahman et al. (2003) reported BS production yield of 1.67 g/L and 4.9 g/L by mixed consortia, respectively. There is an evident difference of an order of magnitude in the BS production yield and this is probably due to our consortium being totally different in composition or the initial inoculum amount being substantially different (we started with a total inoculum of 10^7^ CFU). Our consortium composed mainly by members of the Rhodobacteraceae, Rhodospirillaceae, Shewanellaceae, Alcanivoracaceae, Halomonadaceae, Oceanospirillaceae and Pseudomonadaceae families (**Figure 2**), whereas Rahman et al. (2003) used a consortium of five isolates (*Micrococcus* sp. GS2-22, *Bacillus* sp. DS6- 86, *Corynebacterium* sp. GS5-66, *Flavobacterium* sp. DS5-73 and *Pseudomonas* sp. DS10-129), and Darvishi et al. (2011) used a consortium of two isolates *E. cloacae* and *Pseudomonas* sp.. In all cases, the common denominator is the *Pseudomonas* family members that are well-known hydrocarbon degraders and BS producers. Rahman et al. (2003) also observed that “when oil degraders were introduced individually, the amount of surfactant production was more when compared to the production of surfactant by mixed bacterial consortium,” which is similar to our results. This indicates that there may be a competition between the bacteria for nutrient substrate. However, BS production by the mixed bacterial consortium reported here has not been reported earlier.

When single *A. borkumensis SK2* strains E8Y, E4D, E4F where incubated with CO as the sole carbon source at 14°C the RL production yield increased up to 50 ± 20 mg/l. This represented a more than 100% increase compared to the concentration that complex marine consortia E8 and EB8 produced. SL concentration remained low at 20 mg/l. *Alcanivorax* is known for its glycolipid production as well as hydrocarbon degrading capability (Yakimov et al., 1998; Golyshin et al., 2003). It has been reported in the literature that mainly laboratory strains of *Pseudomonas aeruginosa* (a well described class II pathogen and RL producer) are used to produce yields of 10–20 g/l of RLs, whereas SLs are already produced by manufacturers using *Candida bombicola* yeasts with yields greater than 100 g/l (Wei et al., 2005; Marchant and Banat, 2012; Banat et al., 2014). Daniel et al. (1998) reported a surprisingly high yield (422 g/l) of SLs production by *Candida bombicola ATCC 22214.* The BS yields obtained by marine BS producers and reported here are at least one order of magnitude lower compared to the ones in literature (Rahman et al., 2003; Darvishi et al., 2011).

Most of the published works, study the time course of BS production kinetics in optimal media and substrate conditions, using BS producers whereas in our study we explore the BS production yield by consortia from the marine environment, which produce BS and consume CO.

### Best Hydrocarbon Degrader Strain for Bioaugmentation in Open Sea?

The ESP-A strain, *Paracoccus marcusii* obtained from marine sediment, produced low amounts of BS, ∼20 mg/l of each glycolipid, *while keeping its biomass trapped in the oil phase (CO).* This observation is particularly interesting from a bioremediation point of view. If this strain is used for bioaugmentation purposes, no dilution effects are expected due to sea currents. *Paracoccus marcusii* strains have been reported present in hydrocarbon contaminated areas as general hydrocarbon degraders (Harker et al., 1998; Chaerun et al., 2004), as a promising polyaromatic hydrocarbon (PAH) degrader (Pouli et al., 2008) as well as carotenoid producer (Hirschberg and Harker, 1999). In oil spills, where PAHs are the predominant compounds, bioaugmentation with *Paracoccus marcusii* strains maybe an optimal choice.

### Effect of Substrate Type and Concentration on BS Production

The BS production by *A. borkumensis SK2* strain (E8Y) was investigated for possible enhancement through the use of alternative media. In particular, the tested media were: the substrate hydrocarbon concentration was modified from zero to 0.5% with the use of glucose as the alternate carbon source. No significant increase in the BS concentration was observed by E8Y. This strain when fed with glucose (either entirely 0.5% w/v or partially 0.25% w/v) did not increase at all production, on the contrary RL concentration decreased approximately in half.

This may be an indication that the specific strain needs to be stressed by the presence of a non-water soluble substrate (like CO) to produce “only” the required amount of BS, otherwise acts as hydrocarbon degrader alone. Higher concentration was observed in all cases when as carbon source CO or a heavy fraction of CO was used. Interestingly, when a heavy CO fraction is used as the sole carbon source the final product is not contaminated by the substrate as the latter remains as a different phase in the culture broth and it can be readily removed. One of the main issues the BS industry faces, is the final product contamination with substrate impurities (Banat et al., 2014), when a water insoluble substrate is used (i.e., CO). Here, strain E8Y when fed with soluble substrates, like glucose, BS production was not promoted. Although water-soluble substrates may be attractive for processing (clean facilities), the fact that the same substrate can be easily used over and over again and the ability to be readily replaced in one piece, makes HOFs of CO (high in asphaltene concentration) quite attractive.

### Implications in Bioreactor Operation for BS Production

**FIGURE 9:**
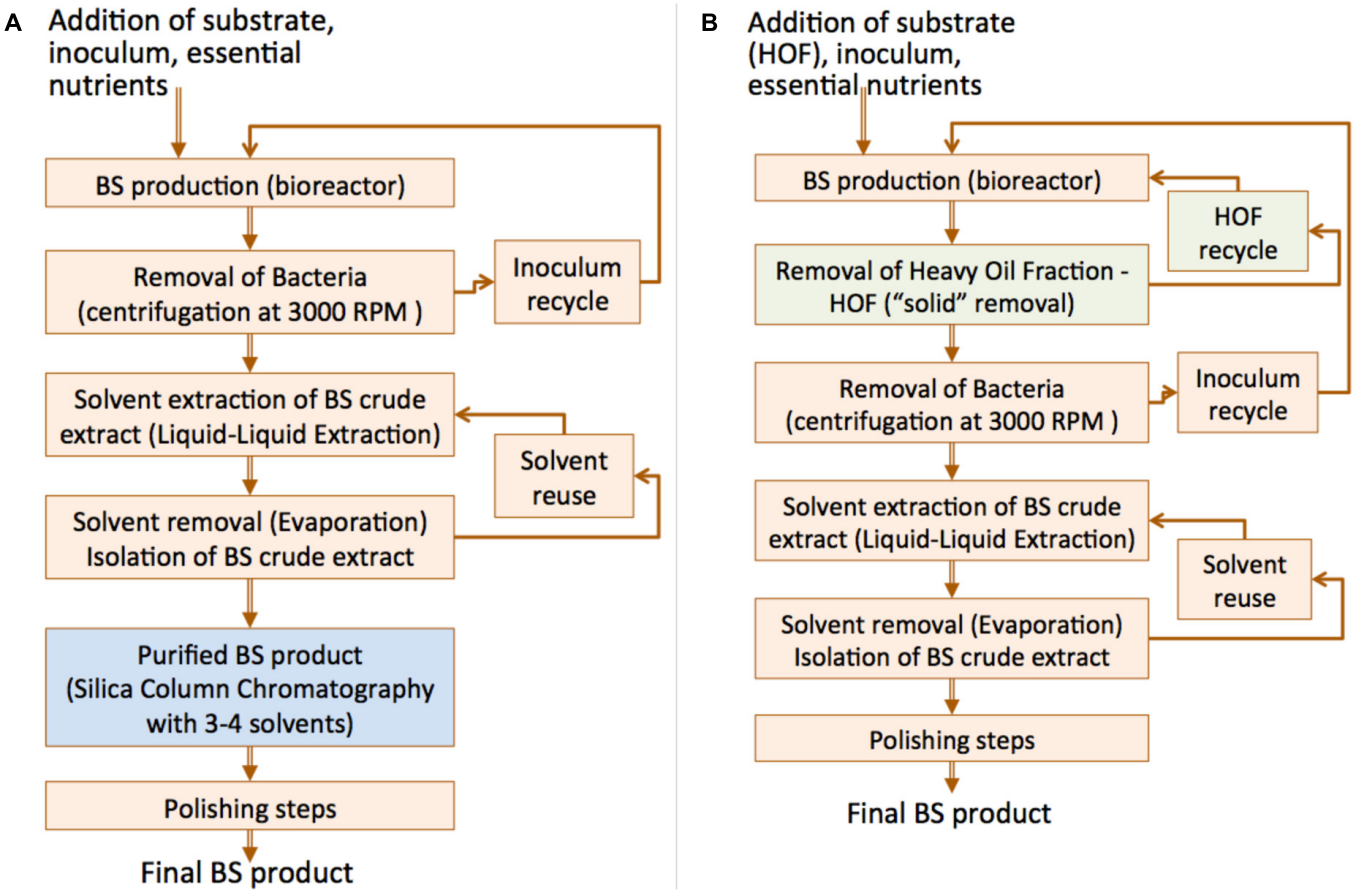
Comparative process flowchart for BS production: **(A)** Typical substrates (i.e., crude oil) that cannot be readily removed from the culture broth and hence the chromatographic separation step is required; **(B)** Use of HOF (rich in asphaltenes) which can be readily removed from culture broth, overcomes the requirement for chromatographic separation step

The above observations lead us to consider the case of bioreactor configuration/operation for BS production using a heavy CO product as carbon source. The latter stresses the marine bacterial isolates to produce BS without being able to solubilize it. Thus, the extraction of the RLs from the aqueous phase is easy and no purification of the BS oil extract is necessary (silica gel column chromatography) as no substrate is dissolved in this case. A comparative process flowchart is given in **Figure 9**.

### Interfacial Behavior of the Rhamnolipids-Sophorolipids Lipid Mixture

How much is the “required amount of BSs that enables dissolution -degradation of the CO?” It is known that addition of RL and/or SF increases substantially the solubility of diesel when in concentration higher than the CMC (Whang et al., 2008). In order to answer the above question we made rough calculations on the amount of BS that is required based on its use and interfacial behavior. The calculations were made with the use of RL data since RL has been proven as a very efficient bioremediation agent (Nikolopoulou et al., 2013b) while it increases the cell surface hydrophobicity (Al-Tahhan et al., 2000).

Assuming that RL is utilized as emulsifier around the oil droplets we calculated (using literature data Helvaci et al., 2004) the number of RLs attached to the oil droplet surface using 0.01–1 μl droplet size emulsion of CO (0.5% wt or 6 ml/l) in water. The amount was in the range of 0.1–0.01 mg/l depending on the oil droplet size (the smaller the emulsion droplet size, the higher the amount of RL needed). This amount is very close to the CMC value of RL 0.1–0.04 mM for 0 up to 1 M NaCl environments (Helvaci et al., 2004). Assuming 50% degradation and decrease of the oil droplet size from 1 to 0.5 μl, the amount of RL/l needed to cover the oil droplets interface remains low (0.012 mg/l). In the extreme scenario of 100% degradation of oil droplets (6 ml/l) 0.01 μl size (big interfacial area) the amount increases to 0.9 mg/l, still small quantity compared to the concentration of RL in the culture broth.

Rhamnolipids also attach on the microbial cell surface (Sotirova et al., 2009). Rough calculations show that for 10^7^ cells per ml (at stationary phase) and 1 μm radius (as a nominal value) of the cell, 8 mg/l RLs could be attached on the microbial surfaces. It is quite interesting as, this value is comparable to the concentration of RLs produced by the mixed consortia E8 and EB8. Hence, the assumption that bacteria produce BS at the same rate they consume (bacteria consume the oil droplets with the BS attached) is probably wrong since most of the RL is attached on their cell surface and dissolved in the culture broth, while a very small amount is used for emulsification (0.01–0.1 mg/l) of the hydrocarbons.

## Conclusion

The BS production capability (RLs and SLs) by marine hydrocarbon degraders isolated from Elefsina bay in Eastern Mediterranean Sea was investigated. The best-isolated microbial consortia based on the drop collapse test exhibited a relatively low productivity with average yields in the order of 20 mg/l. In addition isolated strains, consisting mainly of *Alcanivorax* species, performed significantly better in RLs production (50 ± 20 mg/l) in the presence of CO as substrate. A particular strain isolated from sediments, *Paracoccus marcusii,* may be an optimal choice for bioremediation purposes as it produces BSs, degrades hydrocarbons (especially PAHs) and remains trapped in the hydrocarbon phase not suffering from potential dilution effects by sea currents. The HOF of CO emerged as a promising substrate for BS production by marine BS producers as it can be easily removed from the bioreactor and the BS extract has no impurities by the substrate. Thus, the need for additional column chromatographic separation is bypassed.
